# Effect of the Topology of Carbon-Based Nanofillers on the Filler Networks and Gas Barrier Properties of Rubber Composites

**DOI:** 10.3390/ma13235416

**Published:** 2020-11-28

**Authors:** Shipeng Wen, Rui Zhang, Zongchao Xu, Long Zheng, Li Liu

**Affiliations:** 1 State Key Laboratory of Chemical Resource Engineering, Beijing University of Chemical Technology, Beijing 100029, China; wensp@mail.buct.edu.cn (S.W.); zhangrui971027@163.com (R.Z.); xzcbuct@163.com (Z.X.); 2 Beijing Engineering Research Center of Advanced Elastomers, Beijing University of Chemical Technology, Beijing 100029, China

**Keywords:** gas barrier properties, rubber composites, carbon black, carbon nanotubes, graphene

## Abstract

The topology of nanofillers is one of the key factors affecting the gas barrier properties of rubber composites. In this research, three types of carbon-based nanofillers, including spherical carbon black (CB), fibrous carbon nanotubes (CNTs), and layered graphene (GE) were chosen to investigate the effect of the topological structures of nanofillers on the gas barrier properties of styrene-butadiene rubber (SBR) composites. Results showed that the structure and strength of the filler networks in SBR composites were closely associated with the topology of nanofillers. When filled with 35 phr CB, 8 phr CNTs, and 4 phr GE, the SBR composites had the same strength of the filler network, while the improvement in gas barrier properties were 39.2%, 12.7%, and 41.2%, respectively, compared with pure SBR composites. Among the three nanofillers, GE exhibited the most excellent enhancement with the smallest filler content, demonstrating the superiority of two-dimensional GE in improving the barrier properties of rubber composites.

## 1. Introduction

The application of nanofillers in polymer composites has promoted the development of polymer composites in various fields [1,2,3,4,5]. Rubber is a kind of special polymer composites with high elasticity, which is widely used in tires, conveyer belt, latex products, and so on [6,7,8]. It is generally accepted that nanofillers are one of the indispensable components for preparing rubber composites with high performance. After the introduction of nanofillers into rubber matrix, the mechanical properties, mass transfer properties, dynamic properties, and other properties of rubber composites could be significantly improved [9,10,11,12]. In some special application areas, such as tire inner liners, sealing applications, and chemical protective equipment, etc., more attention was paid to the barrier properties of rubber composites. However, due to the low glass transition temperature of rubber materials and weak interaction between rubber macromolecules, the barrier properties of rubber materials were quite low. In order to improve the barrier performance, nanofillers are routinely introduced into rubber matrix to construct a three-dimensional filler network and form a physical barrier to prevent the penetrating of diffusing molecules, resultingly extending the infiltration pathway of diffusing molecules and enhancing the barrier properties of the rubber composites.

Different types of nanofillers have been used to improve the barrier properties of rubber composites [13,14,15,16,17,18]. According to the topology, these nanofillers could be classified into three categories: (a) spherical nanofillers, such as carbon black (CB) and silica; (b) fibrous nanofillers, such as carbon nanotubes (CNTs) and other fibers; (c) layered nanofillers, such as clay and graphene (GE). Among these nanofillers, spherical CB, fibrous CNTs, and layered GE all belong to carbon-based nanofillers and have the same chemical compositions, but their topologies are obvious different. The application of CB in rubber composites has played an important role for more than one hundred years [19]. In recent years, CNTs and GE have aroused a great research interest in the field of rubber composites due to their functional properties as well as large specific surface area and high aspect ratio [20,21]. Previous studies found that CB, CNTs, and GE had a great impact on the mechanical properties, electrical and thermal conductivity, and fatigue properties of rubber composites, due to the different topological structure of fillers [22,23,24,25]. However, these works did not focus on the comparison of the topology of three different types of fillers and their effect on the gas barrier properties of rubber composites. In addition, most the current researches about the barrier properties of rubber composites were based on single type of filler or two types of fillers [26,27,28,29,30,31,32,33]. For example, Amerongen’s research showed that the gas barrier properties of natural rubber composites were improved by 30% after the introduction of 50 phr (parts per hundreds of rubber) CB [31]. Cadambi et al. [32] found a 33% reduction in permeability after the introduction of 3.5 phr CNTs into hydrogenated nitrile rubber composites. Varghese et al. [33] found the helium leak rate in acrylonitrile butadiene rubber was decreased by 40% in presence of 3 phr GE. The above reports demonstrated that the CB, CNTs, and GE all exhibited contribution on the gas barrier properties of rubber composites. However, there are still few reports focusing on the influence of the topology of nanofillers on the gas barrier properties of rubber composites based on the similar rubber matrix.

In fact, the topology of the nanofillers has an important impact on the morphology of filler aggregations and structure of filler networks, which were closely related to the barrier properties of rubber composites. Therefore, it is necessary to reveal the correlations between the topology of fillers and gas barrier properties of rubber composites.

For this purpose, CB, CNTs, and GE were chosen as the reinforcing fillers. Styrene-butadiene rubber (SBR) was chosen as the host rubber matrix, due to its low cost, good processing performance, excellent elasticity and its widely application in sealings. After the introduction of CB, CNTs, and GE into SBR matrix, the microstructure and gas barrier performance of the obtained composites had been investigated.

## 2. Materials and Methods

### 2.1. Materials

SBR (1502) was provided by Qilu Petrochemical Co., Ltd. (China). CB (N330) with a BET surface area of 71–85 m^2^/g and an average particle size of 30 nm, was purchased from Cabot Chemical (Tianjin) Co., Ltd. CNTs (FT7000) with a BET surface area of 200–300 m^2^/g, an average tube diameter of 7–11 nm, and an average length of 5–20 μm, was purchased from Cnano Technology. GE (LKR4845) with a BET surface area of 400–550 m^2^/g, an average number of layers of 4–6, and an average lateral size of less than 10 μm, was purchased from Qingdao Lankai New Material Technology Co. (Qingdao, China), Ltd. Zinc oxide (ZnO), stearic acid (SA), N-(1-methylethyl)-N’-phenyl (4010NA), N-Cyclohexyl-2-benzothiazolesulfenamide (CZ) and sulfur were all commercially available. All nanofillers and additives were used without any other treatment.

### 2.2. Sample Preparation

SBR composites filled with different contents of CB, CNTs, and GE were prepared by mechanical compounding method. The process of preparing CB/SBR composites was as follows. Firstly, raw SBR (100 phr) was masticated on an open mill for 2 min. Then, the additives including ZnO (2 phr), SA (1 phr) and 4010NA (2 phr) were consecutively added and mixed for another 2 min. Subsequently, 10–40 phr CB were added and mixed for 5 min. Furthermore, CZ (2 phr) and sulfur (2 phr) were added, then CB/SBR compound was obtained. Finally, the compound was vulcanized to obtain CB/SBR composite at the conditions of 160 °C and 15 MPa for an optimum cure time. Similarly, the SBR composites filled with 2–14 phr CNTs or 1–7 phr GE were prepared by the same procedures. For simplicity, the composites were named as CB/SBR-x, CNTs/SBR-y, GE/SBR-z, where x, y, and z represent the contents of CB, CNTs and GE, respectively, in 100 phr of the SBR matrix. For example, GE/SBR-4 denotes that 4 phr GE was contained in the SBR composites.

### 2.3. Characterization

The morphology of the nanofillers and SBR composites were observed by scanning electron microscopy (SEM, S4800, Hitachi, Japan). The elementary compositions of the nanofillers were surveyed through X-ray photoelectron spectroscopy (XPS, ESCALAB 250, Thermo Electron Corporation, Waltham, MA, USA). The crystal structure of the nanofillers were analyzed by X-ray diffraction (XRD, D/Max2500, Rigaku Corporation, Akishima, Japan). The degree of disorder and defects of the nanofillers were investigated through Raman spectroscopy (Renishaw inVia confocal microscope, Renishaw, England). The dispersion of nanofillers in SBR composites were observed by transmission electron microscopy (TEM, G2 20 S-TWIN, FEI Corporation, Hillsboro, OR, USA). Filler networks were analyzed by rubber processing analyzer (RPA) (RPA 2000, Alpha Technologies Corporation, Akron, OH, USA) at a frequency of 1 Hz at 60 °C. Curing characteristics of SBR composites were tested by an oscillating disc rheometer (Model MR-C3, Beijing Ruida Yuchen Instrument Co., Ltd., Beijing, China). Crosslinking density was tested by nuclear magnetic resonance spectroscopy (VTMR20-010V-1, Suzhou Niumag Corporation, Suzhou, China) The restriction effect of nanofillers on SBR macromolecules were investigated through dynamic mechanical analysis (DMA, VA3000, 01-dB-Metravib Corporation, Paris, France) in the tension mode at a frequency of 10 Hz and a strain of 0.3%. The mechanical properties were tested using a tensile test machine (CTM4104, SANS, Shenzhen, China). The tensile and tear tests were carried out according to ISO 37: 2005 and ISO 34-1: 2004, respectively. Five samples were tested for each composite. The permeability coefficient of nitrogen in SBR composites were tested via a gas permeability-measuring apparatus (VAC-V2, Jinan Languang Electromechanical Technology Co., Ltd., Jinan China) at 23 °C. The thickness and diameter of the samples were about 1 mm and 8 cm, respectively.

## 3. Results and Discussion

### 3.1. The Morphology and Structural Characteristics of CB, CNTs and GE

**Figure 1:**
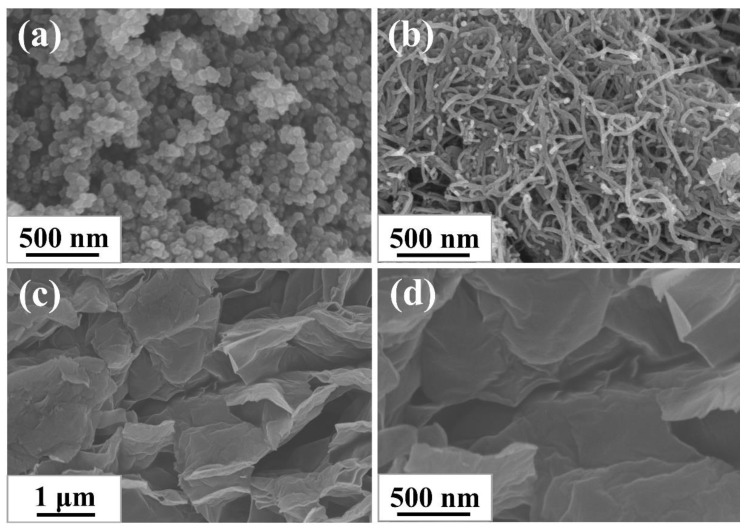
SEM images of (**a**) CB; (**b**) CNTs, and (**c**,**d**) GE.

The morphologies of CB, CNTs, and GE were characterized via SEM to survey their topological structure. As shown in Figure 1a–d, three types of nanofillers all existed in the form of aggregations: 0-dimensional ellipsoid CB particles were fused together, 1-dimensional tubular CNTs particles were entangled and bonded together, and 2-dimensional layered GE particles were stacked together. The features of different morphology of aggregations determined the different structure of filler networks constructed in rubber composites.

**Figure 2:**
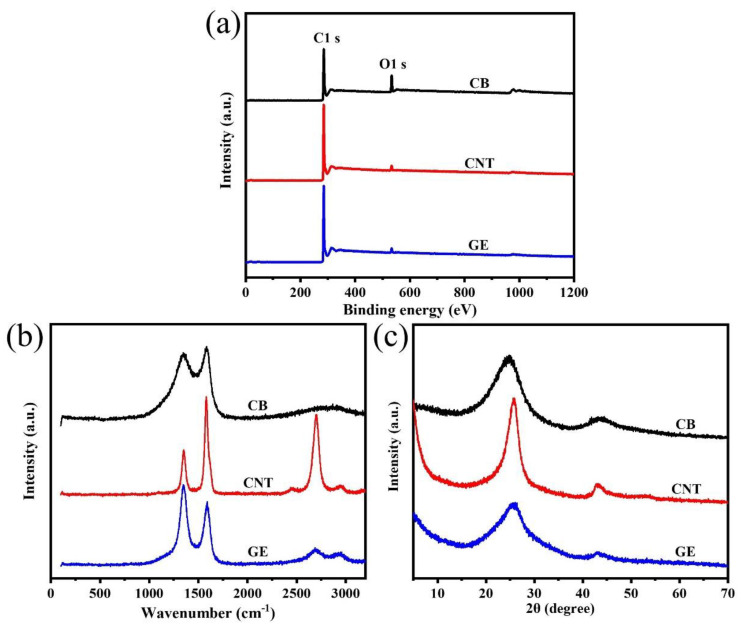
(**a**) XPS survey spectra; (**b**) Raman spectra, and (**c**) XRD patterns of CB, CNTs, and GE.

The XPS survey spectra of CB, CNTs, and GE in Figure 2a show that the main components of the three types of fillers were carbon atoms, while also containing a small amount of oxygen atoms. The presence of oxygen atoms was due to the oxygen-containing groups (such as carbonyl and carboxyl groups) presenting on the surface of the nanofillers [34]. The percentage of oxygen atoms in CB, CNTs, and GE was 9.04%, 2.25%, and 2.18%, respectively, indicating the oxidation degree of CB was higher than that of CNTs and GE.

Raman spectra of CB, CNTs, and GE in the Figure 2b show the typical D band (1350 cm^−1^) and G band (1580 cm^−1^) of carbon materials. The D band represents the defect or disorders induced in the graphitic lattice, while G band is related to the stretching of carbon sp2 structure [35]. The integral intensity ratio of D band and G band (I_D_/I_G_), which was used to characterize the degree of defect of the nanofillers, was 2.3, 0.6, and 1.5 for CB, CNTs, and GE, respectively, indicating more defects in CB than other nanofillers.

The XRD patterns in Figure 2c show two distinct diffraction peaks around 25° and 43°, corresponding to (002) and (110) crystal plane. The (002) peaks for CNTs and GE were at 2θ = 25.7°, while for CB was at 2θ = 24.8°. According to Bragg’s law, the d spacings of CB and CNTs (GE) were 3.6 Å and 3.5 Å, respectively. The d spacing of CB was slightly larger than that of CNTs and GE, caused by more oxygen-containing groups on the surface of CB.

The above characterizations confirmed that the chemical compositions of CB, CNTs, and GE are basically the same, while the morphology and structural characteristics are quite different, which have an important impact on the microstructure and gas barrier properties of SBR composites.

### 3.2. Filler Networks in SBR Composites

**Figure 3:**
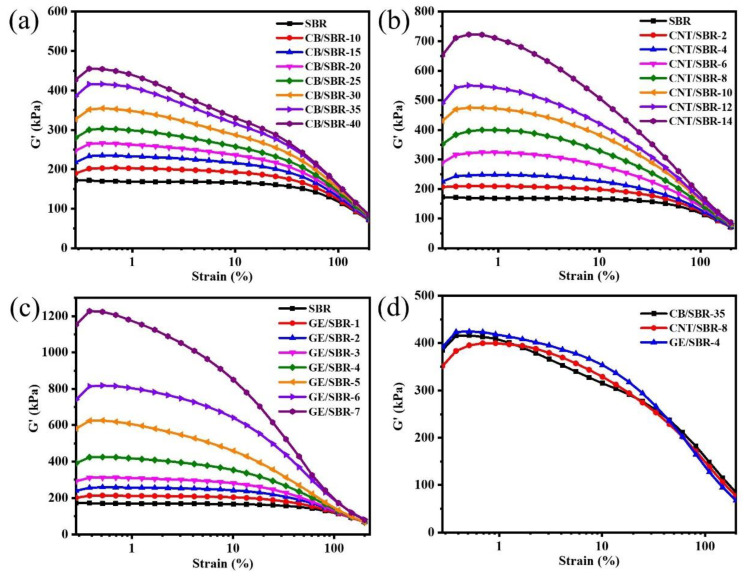
Storage modulus versus dynamic strain of SBR compounds filled with different contents of (**a**) CB; (**b**) CNTs and (**c**) GE; (**d**) storage modulus versus dynamic strain of CB/SBR-35, CNTs/SBR-8, and GE/SBR-4 compounds.

Filler networks play a vital role in preventing the penetration of gas molecules through rubber composites. Therefore, RPA was used to analyze the filler networks composed of CB, CNTs, or GE in SBR composites. In Figure 3a–c, with the increase in the filler contents, the storage modulus (G’) of CB/SBR, CNTs/SBR, and GE/SBR composites gradually increased, indicating the filler networks in SBR composites were gradually strengthened [36]. However, the three types of fillers had different enhancement capabilities to the G’ of SBR composites. Although the content of CB was much larger than that of CNTs and GE, the G’ of CB reinforced SBR composites were lower than that of CNTs or GE reinforced composites. As shown in Figure 3d, the G’ values of SBR composites containing 35 phr CB, 8phr CNTs, or 4 phr GE were almost the same, suggesting the strength of the filler network in the three SBR composites were basically the same. This phenomenon indicated that the topological structure of the nanofillers had a close impact on the construction of the filler network in rubber composites. In fact, the aspect ratio of spherical CB is much smaller than that of tubular CNTs and layered GE. CNTs or GE nanofillers could form a dense filler network at lower filler content, while CB nanofillers only overlapped each other to form a filler network only at a high filler content.

### 3.3. Curing Characteristics and Crosslinking Density of SBR Composites

**Figure 4:**
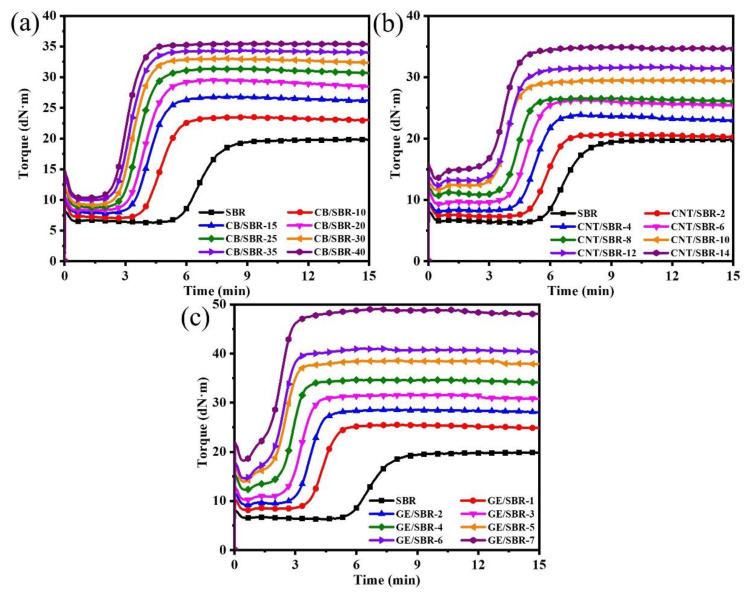
Curing characteristics of SBR compounds filled with different contents of (**a**) CB; (**b**) CNTs and (**c**) GE.

Curing is an important step in the process of preparing rubber products. After curing, the linear rubber macromolecules were crosslinked to form a three-dimensional cross-linked network, which endowed the rubber composites unique high elasticity. Figure 4 shows the curing characteristics curves of SBR composites filled with different contents of CB, CNTs, and GE. In Figure 4, it is clear to observe that the variation tendencies of the three types of composites were basically similar. The scorch time and optimum curing time were shortened after introducing nanofillers into the SBR matrix regardless of the topology of nanofillers, indicating the curing reaction was activated by the nanofillers. The minimum torque (M_L_), maximum torque (M_H_), and torque difference (ΔM) between M_H_ and M_L_ increased constantly with increasing filler content. M_L_ is mainly related to the filler–filler interaction in the compound. The M_L_ values of all the compounds increased constantly with increasing filler content owing to the enhanced filler networks. M_H_ is mainly affected by filler–filler interactions, filler–rubber interactions, and rubber–rubber interactions. As the filler content increases, the M_H_ value also showed an upward trend. This was because the increase in nanofiller content not only strengthened the filler network, but also increased the contact points between the nanofillers and the SBR macromolecules, thus forming a stronger filler–rubber interaction. In addition, the ΔM was generally proportional to the crosslinking density of the composites. When the filler content increased, the strengthened filler–rubber interactions formed more physical crosslinking points, resulting the crosslinking density of the composites gradually increased. For example, the ΔM of CB/SBR-35, CNTs/SBR-8, and GE/SBR-8 were 24.44, 15.92, and 22.33, respectively, while the crosslinking density of CB/SBR-35, CNTs/SBR-8, and GE/SBR-8 were 3.71, 2.42, and 3.1 × 10^−4^ mol/mL, respectively. High crosslink density is beneficial to the improvement of mechanical properties and barrier properties.

### 3.4. The Microstructure of SBR Composites

**Figure 5:**
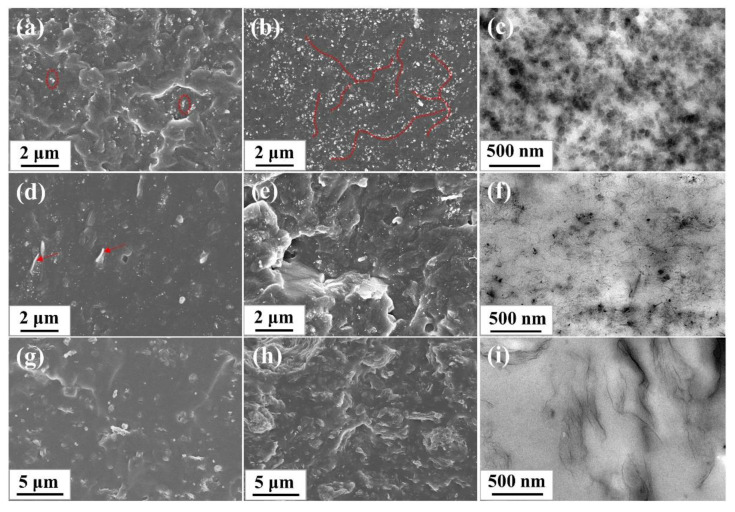
SEM images of different SBR composites: (**a**) CB/SBR-10; (**b**) CB/SBR-35; (**d**) CNTs/SBR-2; (**e**) CNTs/SBR-14; (**g**) GE/SBR-1; (**h**) GE/SBR-7. TEM images of (**c**) CB/SBR-35; (**f**) CNTs/SBR-8, and (**i**) GE/SBR-4 composites.

SEM and TEM was used to observe the dispersion and distribution of the nanofillers in the rubber matrix. The SEM and TEM images of SBR composites were shown in Figure 5. For CB/SBR composites, when the content of CB was gradually increased from 10 phr to 35 phr, the uniformly distributed CB gradually aggregated to form continuous chains (indicated by the red line), and further to form three-dimensional overlapping networks. For CNTs/SBR composites, when the content of CNTs was 2 phr, some CNTs were exposed outside the rubber matrix (indicated by the red arrow), suggesting the weak interface between CNTs and SBR matrix. When the content of CNTs increased, the agglomeration of CNTs became more serious. Especially when the content of CNTs reached 14 phr, some voids appeared in the SEM image. For GE/SBR composites, when the content of GE was 1 phr, the morphology of fractured surface was flat. After increasing the contents of GE, the fractured surface of the composites became rough, and GE stacked to form obvious aggregates that were wrapped in the SBR matrix. The difference in the morphology of the filler aggregates originated from the difference in topology of the nanofillers. In TEM images, the dark gray particles or lines in TEM images were nanofillers, and the light gray area were SBR matrix. In Figure 5c, it is clearer to see the long-chain structure and overlapping networks formed by the small CB aggregates. In Figure 5f, the curled CNTs were entangled and bonded with each other in SBR matrix. In Figure 5i, layered GE stacked and connected to each other to form a filler network. Due to the high aspect ratio of CNTs and GE, a dense filler network was more easily to form in SBR matrix at a low filler content.

**Figure 6:**
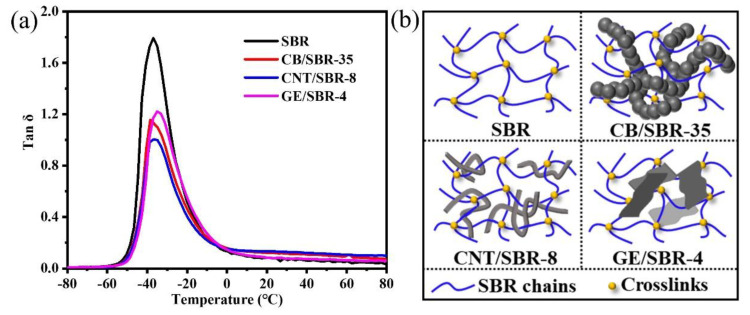
(**a**) Loss factor (tan δ) versus temperature of and (**b**) schematic diagram of the filler networks of SBR, CB/SBR-35, CNTs/SBR-8, and GE/SBR-4 composites.

Furthermore, DMA was used to analyze the restriction effect of nanofillers on SBR macromolecules. Figure 6a shows the peak values of tan δ of the composites were reduced by different degrees, after addition of different nanofillers into the SBR matrix. The CNTs filled SBR composites exhibited the smallest peak value of tan δ among the three composites. The small peak value of tan δ indicates the strong restriction effect of the fillers on the SBR macromolecules [37,38]. Therefore, CNTs had the strongest restriction effect on the SBR macromolecules among the three fillers, when the strengths of the filler networks were the same. In addition, the glass transition temperature (Tg) of pure SBR was about −36.9 °C, which was close to the value in the literature [39]. After the introduction of CB or CNTs, the Tg of CB/SBR-35 and CNTs/SBR-8 had almost no change when compared with pure SBR. This phenomenon was consistent with the literature [40,41], while the Tg of GE/SBR-4 increased to about −35 °C. This was because the large GE sheets in the SBR matrix provided more physical crosslinks [39].

According to the above analysis of microstructure, the schematic diagrams of the filler networks of SBR, CB/SBR-35, CNTs/SBR-8, and GE/SBR-4 composites were demonstrated in Figure 6b. For pure SBR composites, although the introduction of crosslinking bonds could restrain the movement of rubber macromolecules in a certain degree, the SBR rubber macromolecules still possessed strong mobility. Therefore, there was still a large amount of free volumes in SBR composites. After the introduction of 35 phr CB into SBR matrix, the continuous CB chains interspersed into the crosslinking networks, thus reducing the mobility of SBR macromolecules. When 8 phr CNTs was filled, due to the slender and tubular characteristics of CNTs, it was not only easy to intersperse into the crosslinking network, but also entangle on the rubber macromolecules. When 4 phr GE was filled, some rubber macromolecules were absorbed on the surface of GE sheets, resulting GE sheets difficult to intersperse into the crosslinking networks because of its large lateral size. Therefore, CNTs exerted strongest restraint on the mobility of the SBR macromolecules when the strength of the filler networks was the same. The restriction effect of the filler networks on the rubber macromolecules reduced the free volumes in the composites, which was beneficial to the improvement of the gas barrier properties of the composites. Meanwhile, the filler networks formed by the nanofillers with different topologies played a more important role in preventing the penetration of the gas molecules.

### 3.5. Mechanical and Thermal Properties of SBR Composites

**Figure 7:**
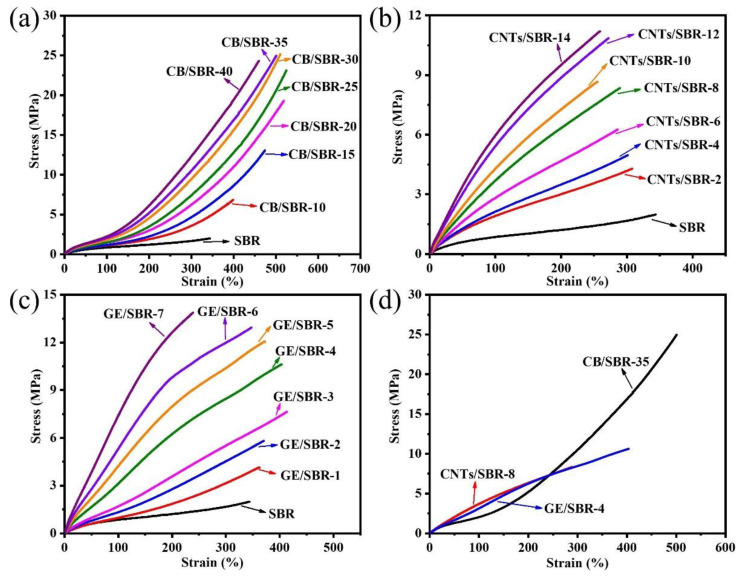
Strain-stress curves of SBR composites filled with different contents of (**a**) CB; (**b**) CNTs and (**c**) GE; (**d**) strain-stress curves of CB/SBR-35, CNTs/SBR-8 and GE/SBR-4 composites.

**Figure 8:**
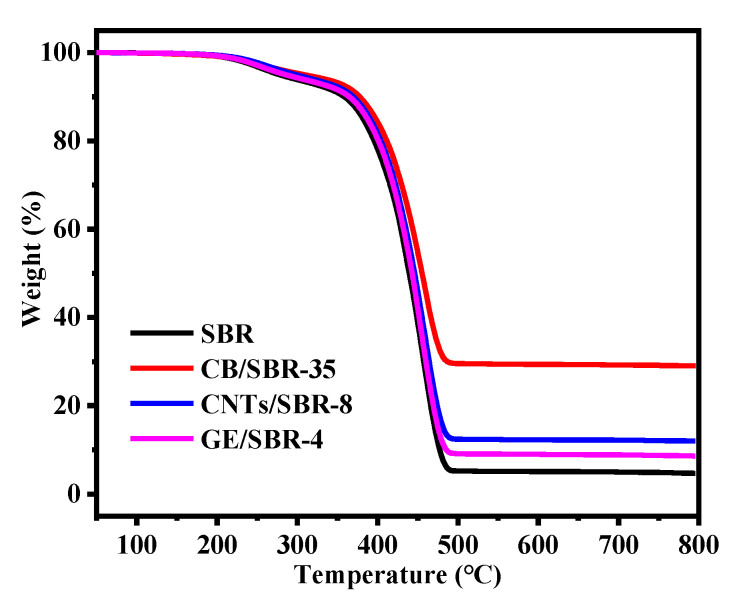
TGA curves of SBR, CB/SBR-35, CNTs/SBR-8, and GE/SBR-4.

The stress-strain curves of SBR composites reinforced by different contents of CB, CNTs, and GE were shown in Figure 7a–c. The results showed that the modulus at 100% strain increased with the increase in the nanofiller contents. The increase was attributed to the enhanced filler networks which were conducive to transferring the stress from SBR macromolecules to filler networks. Figure 7d shows the strain-stress curves of the different SBR composites with the same strength of filler networks, and the mechanical properties were displayed in Table 1. CNTs/SBR-8 and GE/SBR-4 composites exhibited similar tensile behaviors, which were significantly different from that of CB/SBR-35 composite. This was caused by the different structure of filler networks formed by the three types of topological fillers. Among the three composites, CNTs/SBR-8 composite possessed the highest modulus at 100% strain and the smallest elongation at break, reflecting that CNTs had the strongest restriction effect on SBR macromolecules. As for CB/SBR-35 and GE/SBR-4 composites, although the filler network formed by 35 phr CB had a stronger restriction on SBR macromolecules than the filler network formed by 4 phr GE, the continuous CB chains were easily broken, and SBR macromolecules would slip on the surface of CB during the stretching process. Thus, CB/SBR-35 composite possessed higher elongation at break and modulus at 300% strain than GE/SBR-4 composite, while GE/SBR-4 composite possessed higher modulus at 100% strain than CB/SBR-35 composite. Moreover, the tear strength of CB/SBR-35 was much higher than CNTs/SBR-8 and GE/SBR-4. This was attributed to the dissipation of energy during stretching caused by the continuous slippage of SBR macromolecules on the surface of CB. It is also noticeable that the hardness of GE/SBR-4 was much higher than CB/SBR-35 and CNTs/SBR-8. This is because the large GE sheets could adsorb more SBR macromolecules and form physical crosslinking points, thereby making GE/SBR-4 possess higher hardness. In addition, the TGA curves of pure SBR, CB/SBR-35, CNTs/SBR-8 and GE/SBR-4 composites were displayed in Figure 8. After analyzing the curves in Figure 8, it is found that the thermal stability of the obtained composites was mainly related to the contents of fillers. The more filler contents, the better the thermal stability of the composite.

### 3.6. Gas Barrier Properties of SBR Composites

**Figure 9:**
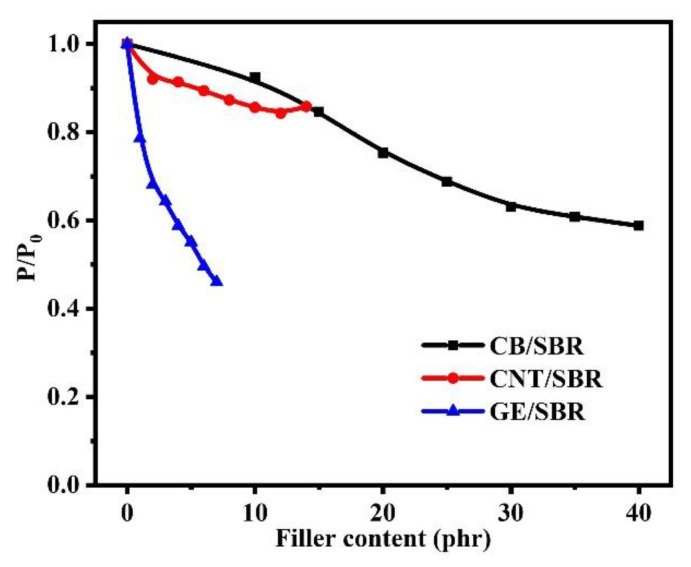
The relative permeability coefficients of SBR composites filled with different contents of CB, CNTs and GE.

The gas barrier properties of rubber composites were strongly dependent on the filler networks in rubber composites. The relative permeability coefficients (p/p_0_), which are the ratio of the permeability of SBR composite to the permeability of pure SBR, were shown in Figure 9. The results show that the gas barrier properties of the SBR composites were all improved to a certain extent after the addition of nanofillers. However, the composites reinforced by different topology of nanofillers showed different trend in improvement of gas barrier properties. For CB/SBR composites, when CB content was lower than 30 phr, the relative permeability coefficients of the composites gradually decreased as the CB content increased. When CB content exceeded 30 phr, the relative permeability coefficients of the composites tended to be stable. For CNTs/SBR composites, when CNTs content was lower than 12 phr, the relative permeability coefficients of the composites gradually decreased as the CNTs content increased. When CNTs content reached 14 phr, the relative permeability coefficient of the composites increased instead, which was caused by the severe aggregation of CNTs. It is noted that the composites filled with 2 phr CNTs and 10 phr CB possessed almost equal relative permeability coefficient, demonstrating that one-dimensional CNTs with high aspect ratio had more advantage than spherical CB in improving the gas barrier properties rubber composites at low filler content. For GE/SBR composites, the relative permeability coefficients gradually decreased with the increase in GE content. The relative permeability coefficient of SBR composites filled with 1 phr GE was lower than those of SBR composites filled with 12 phr CNTs or 15 phr CB, revealing the superiority of GE than CNTs and CB in improving the barrier properties of rubber composites.

As for CB/SBR-35, CNTs/SBR-8, and GE/SBR-4 composites, the gas barrier properties of the three composites were increased by 39.2%, 12.7%, and 41.2%, respectively, compared with pure SBR. GE/SBR-4 composite exhibited the best gas barrier properties among the three composites with the same strength of the filler network. This result was also related to the topology of the nanofillers and the structure of filler networks. Two-dimensional GE had a high aspect ratio, and could form a more complete filler network at a low filler content, thus prolonging the pathways of gas molecules and resulting in high gas barrier properties.

## 4. Conclusions

In this study, three carbon-based nanofillers with different topologies, including CB, CNTs, and GE, were selected to reveal the effect of topology of nanofillers on the gas barrier properties of rubber composites. The results showed that CB formed a dense filler network only at a high filler content, while CNTs or GE formed a dense filler network at a low filler content. The SBR composites had the same strength of the filler networks, when filled with 35 phr CB, 8 phr CNTs, or 4 phr GE, respectively. However, the structures of the three filler networks were obvious different, thus resulting in different improvement effect on gas barrier properties. The gas barrier properties of the three composites were increased by 39.2%, 12.7%, and 41.2%, respectively, compared with pure SBR composite. The results showed that the two-dimensional layered GE had more advantages in improving gas barrier performance.

## Figures and Tables

**Table 1:** The mechanical properties of pure SBR, CB/SBR-35, CNTs/SBR-8, and GE/SBR-4 composites.

Samples	SBR	CB/SBR-35	CNTs/SBR-8	GE/SBR-4
Tensile strength (MPa)	2.0 ± 0.5	25.0 ± 1.8	8.3 ± 1.2	10.6 ± 1.4
Tear strength (kN/m)	7.4 ± 1.6	39.4 ± 3.1	30.7 ± 2.4	32.5 ± 2.9
Elongation at break (%)	344 ± 9	501 ± 18	289 ± 11	403 ± 14
Modulus at 100% strain (MPa)	0.8 ± 0.1	2.0 ± 0.1	3.7 ± 0.2	3.1 ± 0.2
Modulus at 300% strain (MPa)	1.7 ± 0.1	10.5 ± 0.6	--	8.5 ± 0.3
Shore A hardness	48 ± 1	61 ± 1	62 ± 1	66 ± 1
